# Parity and risk of stomach cancer by sub-site: a national Swedish study

**DOI:** 10.1038/sj.bjc.6604283

**Published:** 2008-04-01

**Authors:** S Bahmanyar, M Lambe, K Zendehdel, O Nyrén, P Boffetta, W Ye

**Affiliations:** 1 Department of Medical Epidemiology and Biostatistics, Karolinska Institutet, Stockholm, Sweden; 2 Faculty of Medicine, Golestan University of Medical Sciences, Gorgan, Iran; 3 Cancer Institute Research Centre, Medical Sciences/University of Tehran, Tehran, Iran; 4 International Agency for Research on Cancer, Lyon, France

**Keywords:** stomach cancer, parity, odds ratio, nested case–control study

## Abstract

We investigated stomach cancer risk by anatomic sub-site in relation to parity, as a marker for higher exposure to sex hormones, in a case–control study, nested within a cohort of 2 406 439 Swedish women born in 1925 or later and followed from 1970 or age 30 until emigration, death, any cancer diagnosis, or through 2004, whichever occurred first. We identified 286 cardia and 2498 non-cardia stomach cancer cases with five matched controls for each case. Cross-linkage with the Multi-Generation Register provided information about reproductive history. Using conditional logistic regression models for estimating odds ratios (ORs) and corresponding 95% confidence intervals (CIs), adjusted for education level and occupation, we found no association between any aspect of parity and non-cardia stomach cancer (OR=1.01, 95% CI 0.89–1.15, comparing parous with nulliparous women). However, a 30% risk reduction for postmenopausal cardia cancer (OR=0.7, 95% CI 0.4–1.0) was noted among parous relative to nulliparous women and the risk for premenopausal cardia cancer fell with increasing number of children (*P* for trend=0.04). Our results indicate that exposure to female sex hormones does not protect against non-cardia stomach cancer and does not explain male predominance. The observed moderate inverse relationship between parity and cardia cancer may be mediated by non-hormonal factors and warrants further study.

Despite a decreasing trend in incidence, stomach cancer is the fourth most common cancer in the world (Parkin *et al*, 2005). In most areas its incidence is about twofold higher among men than among women, apparently not entirely explained by known risk factors such as, for example, *Helicobacter pylori* infection and tobacco smoking (Nyren and Adami, 2002). A male preponderance (Furukawa *et al*, 1982a) and a protective effect of female hormones have been reported for stomach cancer in rat experimental models (Furukawa *et al*, 1982b). As functional oestrogen receptors alpha (Wu *et al*, 1990; Singh *et al*, 1997) and beta (Taylor and Al-Azzawi, 2000; Matsuyama *et al*, 2002) exist in human stomach mucosa, it has been hypothesised that sex hormones, notably oestrogens, protect women against stomach cancer (Sipponen and Correa, 2002).

Pregnant women have markedly elevated serum levels of some hormones, including oestrogens (Cunningham *et al*, 2001). The hyper-oestrogenic state increases continually through pregnancy, and finally oestrogen production rises more than 1000-fold. Few studies have investigated stomach cancer risk in relation to reproductive factors and results have been inconsistent (Miller *et al*, 1980; Kvale *et al*, 1994; La Vecchia *et al*, 1994; Palli *et al*, 1994; Inoue *et al*, 2002; Kaneko *et al*, 2003; Frise *et al*, 2006). Although risk profiles for cardia and non-cardia stomach cancer differ, only one study (Frise *et al*, 2006) has examined risks separately.

We used information retrieved from the national Swedish Multi-Generation and Cancer registers to conduct a case–control study nested within a large cohort of Swedish women for any association between parity and stomach cancer risk by anatomic sub-site.

## MATERIALS AND METHODS

The cohort was based on the Swedish Multi-Generation Register, which includes index persons born in 1932 or later and alive in 1961, with links to their parents. Mothers born in 1925 or later and alive, and registered in the population register in 1947, when National Registration Numbers (NRNs) were introduced, or later, constituted our cohort. NRN, a unique identifier assigned to all Swedish residents at birth or upon immigration, was used for unambiguous linkages with several nationwide registers. The Historic Population, Migration, and Causes of Death Registers were used to obtain information about marital status and dates of emigration and death, respectively. The Swedish Cancer Register, established in 1958 and more than 98% complete (Mattsson and Wallgren, 1984; Ekstrom *et al*, 1999), contains individual data on all newly diagnosed malignant tumours in Sweden, coded to the 7th revision of International Classification of Disease (ICD7). Stomach cancer (code 151) was further classified as cardia and non-cardia cancer using a supplementary code introduced in 1970. Therefore, and since stomach cancer rarely occurs at young ages, the follow-up forming the base for the present nested case–control study started on 1 January 1970 or thereafter at age 30 years. After excluding subjects with any cancer diagnosed before start of follow-up, 2 406 439 women were enrolled. Follow-up continued until the date of diagnosis of any cancer, emigration, death, or until 31 December 2004, whichever occurred first. For every observed incident case of stomach cancer, we randomly selected five controls, individually matched on year of birth, alive at diagnosis of their matched cases, and without cancer before selection.

The Swedish Multi-Generation Register provided information on parity and age at first birth. We further linked all cases and controls to the Swedish National Censuses and Education Register in order to obtain the highest occupational class and education level before the time of selection, as markers of socioeconomic status.

### Statistical analyses

We used conditional logistic regression models to estimate odds ratios (ORs) with their corresponding 95% confidence intervals (CIs) as the measure of association between parity or age at first birth and risk. We first estimated crude ORs (inherently adjusted for year of birth) and then multivariately with additional adjustment for highest attained education (in three groups, viz. 9 years comprehensive school or less, upper secondary school (10–12 years), university education (>12 years)) and occupational class (in six groups, viz. manual, non-manual workers, professionals, farmers, self employed, and others). We also considered place of residence (in three regions, south, middle, and north of Sweden) as a possible confounder, but as this did not materially affect our estimates, it was not included in the final models.

We further studied the effect of number of children (1, 2, 3, and >3). Age at first birth was categorised based on the quartile values derived from the entire cohort (<21, 21–24, 25–27, and ⩾28 years). Dose–response relationships were studied only among parous women, and multivariate models mutually adjusted for parity and age at first birth. We used median values of age at first birth for each stratum and also parity as a continuous variable for trend analyses. Women were categorised as pre- or postmenopausal by using age 50 as the cut-off point, based on the approximate median value in several studies (Luoto *et al*, 1994; Gold *et al*, 2001; Rodstrom *et al*, 2003).

As the Swedish Multi-Generation Register only includes offsprings born in 1932 or later, and who were alive in 1961, information about deceased children born around between 1940 (when the mothers in the cohort become sexually mature) and 1961 may be missing. As this could lead to some misclassification, we performed an additional analysis restricted to women born in 1946 or later and thus unlikely to have given birth to children before 1961.

It is well known that unmarried status and living as a single are associated with poorer general health among both men and women, including higher risks for lifestyle-related cancers such as stomach cancer. We therefore reanalyzed data restricted to those who were married at cancer diagnosis (applied also to their corresponding controls) to check if our results were affected by marital status.

The *P*-value of the interaction term in the conditional logistic regression model was used as a test of homogeneity to compare ORs between pre- and postmenopausal women. All statistical analyses were performed using the SAS software version 9.1 (SAS Institute, Cary, NC, USA). This study was approved by the Regional Ethics Committee at the Karolinska Institutet.

## RESULTS

We identified 2498 women with non-cardia stomach and 286 women with cardia cancer between 1970 and 2004, and selected 12 490 and 1430 matched controls, respectively. The mean age of the non-cardia cases was slightly lower (57.0) than that of cardia cancer cases (59.4) (Table 1).

### Non-cardia stomach cancer

Parous women had the same risk for non-cardia stomach cancer as nulliparous women (OR=1.01, 95% CI 0.89–1.15) (Table 2). However, using premenopausal non-cardia stomach cancer as the outcome, parity was associated with reduced OR of borderline significance (OR=0.82, 95% CI 0.65–1.03). The *P*-value of the test of homogeneity of results of pre- and postmenopausal cancers was 0.02. Analyses by parity or age at first pregnancy among parous women did not show any significant variation in ORs. Restriction to married women or to those born in 1946 or later produced no important departure from overall results.

### Cardia cancer

We found a 30% decreased risk of cardia cancer among parous compared with nulliparous women (OR=0.7, 95% CI 0.5–1.1) (Table 3). This inverse association was observed only for postmenopausal cardia cancer (OR=0.7, 95% CI 0.5–1.0), but the trend with parity was significant (*P*=0.04) also for premenopausal cardia cancer (although based on small numbers). No conspicuous variation was observed in relation to age at first pregnancy. Restriction to cases and controls who were married at cancer diagnosis revealed stronger association (OR for cardia cancer overall=0.4, 95% CI 0.2–0.9; for postmenopausal cardia cancer=0.3, 95% CI 0.1–0.9), whereas restriction to women born in 1946 or later produced no noteworthy change, albeit based on very small numbers (data not shown).

Socioeconomic status in our investigation was rather crude, since it combined two different classifications used in, respectively, the 1970 and 1990 censuses. We also repeated the analyses using only 1990 census information because this classification was finer (in eight groups, viz. unskilled manual, skilled manual workers, assistant non-manual, intermediate non-manual employees, professionals, other higher non-manual employees and upper-level executives, self-employed, farmers, and others). The point estimates thus obtained were almost identical to those in the main analyses (data not shown). However, as a considerable number of cases and controls who emigrated or died before 1990 were excluded in these supplementary analyses, we only present the results of analyses using the crude socioeconomic classification.

## DISCUSSION

This nested case–control study gave no indication that pregnancies confer protection against non-cardia stomach cancer, whereas some support for an inverse association between parity and cardia cancer risk emerged.

The large sample size and the population-based design constitute the strengths of this study. Case ascertainment through the nationwide Swedish Cancer Register was virtually complete. There was possible misclassification of exposure due to missed offsprings who died before 1961, but additional analyses restricted to women born in 1946 or later suggested that this is unlikely to have importantly affected the results. However, abortions, miscarriages, and stillbirths were not recorded. This non-differential misclassification may have slightly shifted ORs towards the null value. However, small numbers limit the interpretation of our results for cardia cancer.

An important limitation, as in most register-based studies, is the lack of information on some potential confounding factors such as smoking, a risk factor for stomach cancer, and it is conceivable that mothers smoke less than childless women. However, available data on smoking from other large cohorts of Swedish women do not support this (Gram *et al*, 2005). In corroboration, within our cohort lung cancer incidence among parous compared with nulliparous women gave an age-adjusted relative risk close to unity (1.04, 95% CI 0.97–1.11). Confounding by smoking, therefore, represents an unlikely explanation for the lower risk of cardia cancer among parous women. High body mass index (BMI) and gastroesophageal reflux disease are other risk factors for cardia cancer, and pregnancy is associated with overweight, obesity (Linne *et al*, 2002), and gastroesophageal reflux disease (Cunningham *et al*, 2001). However, any confounding by high BMI and reflux disease should have increased cardia cancer risk among parous women, and so it does not explain the inverse association. Since obesity and gastroesophageal reflux are essentially unrelated to non-cardia stomach cancer, it is unlikely that they have greatly confounded its association with parity.

With adjustment for socioeconomic status, it is improbable that parity is associated with *H. pylori* infection status unless the latter is related to fertility. If a relationship between infection and fertility did exist, the observed inverse association between parity and cardia cancer would be expected only if *H. pylori* infection was linked to increased fertility, a less likely, albeit not totally inconceivable, possibility. However, if infected women were more fertile, parity would be positively associated with risk of non-cardia cancer, contrary to our finding.

As we also lacked information about hereditary predisposition, diet, occupational exposures, and use of non-steroidal anti-inflammatory drugs, factors that are linked to risk of stomach cancer and whose relationship to parity is difficult to predict, we cannot exclude all confounding, nor can we confidently rule out chance as the explanation for the cardia cancer finding given the limited number of cases observed and the multiple testing. Our categorisation of women as pre- or postmenopausal by using age 50 as the cut-off point is subject to misclassification.

Among previous studies of reproductive factors, only one recent Canadian case–control study separately considered cardia and non-cardia stomach cancers (Frise *et al*, 2006 and Table 4); a decreased risk was found for both types among women with over three children compared with nulliparous women. However, the number of exposed cases (>3 pregnancies) was small (14 cardia and 57 non-cardia stomach cancers) and only overall OR estimate was statistically significant.

Other studies of parity and risk of postmenopausal stomach cancer, not considering the anatomical subtypes, found relative risks close to unity among parous compared with nulliparous women (Palli *et al*, 1994; Inoue *et al*, 2002). However, one cohort study (Kaneko *et al*, 2003), using Death Registry data, reported a non-significant inverse association with postmenopausal stomach cancer.

There are inconsistent results regarding parity and stomach cancer risk. Two studies (Kaneko *et al*, 2003; Frise *et al*, 2006) found a suggestive inverse relationship but no significant dose–risk trend, whereas others found either no association (Kvale *et al*, 1994; Palli *et al*, 1994; Inoue *et al*, 2002) or a positive association (one hospital-based case–control study (Miller *et al*, 1980); Plesko *et al*, 1985; La Vecchia *et al*, 1994, the latest two studies including no adjustment for socioeconomic status). We found women with more than one child showing borderline significant 30% risk deficit for postmenopausal cardia cancer compared with those with one child. However, the absence of a significant dose–response trend weighs against a causal inference. On the other hand, there was a significant dose–risk trend for premenopausal cancer, although only one OR estimate among single exposure categories was significant.

Findings on stomach cancer risk and age at first birth have been inconsistent, with previous studies reporting both increased (Palli *et al*, 1994; Kaneko *et al*, 2003) and decreased (La Vecchia *et al*, 1994; Inoue *et al*, 2002) risks among women with higher age at first birth. We found no significant variation in risk of cardia or non-cardia cancer linked to age at first birth.

We knew of no biological rationale why the inverse parity-risk relationship should be limited to cardia cancer. Chance should be the explanation. But if true, the risk reduction seems to be mediated by factor(s) – not necessarily hormonal – that affect cancer development in the cardia and in the rest of the stomach differently. The hypothesis, therefore, that oestrogens play a role in stomach cancer aetiology is given no real support. On the other hand, parity may be an imperfect marker of the sex hormone exposure that is potentially relevant; women are continuously exposed to oestrogen during their fertile life, for up to 40 years, and the pregnancy boosts, although impressive in terms of dose, may be too short-lived or inadequately timed for an appreciable effect on gastric carcinogenesis.

In conclusion, our results point fairly persuasively against a significant inhibitory role of oestrogen in non-cardia stomach cancer. Previous evidence of an inverse association with stomach cancer risk was, at best, only suggestive and with our study weighs towards no association. However, our finding of a significantly decreased risk of cardia cancer among parous, particularly postmenopausal women, warrants further study.

## Figures and Tables

**Table 1:** Characteristics of the study population

	**Non-cardia stomach cancer**		**Cardia cancer**	
	**Cases, *n*** **(%)**	**Controls, *n* (%)**	**Cases, *n* (%)**	**Controls, *n* (%)**
*Age (years)*				
30–39	188 (7.5)	940 (7.5)	10 (3.5)	50 (3.5)
40–49	540 (21.6)	2700 (21.6)	50 (17.5)	250 (17.5)
50–59	728 (29.1)	3640 (29.1)	79 (27.6)	395 (27.6)
60–69	677 (27.1)	3385 (27.1)	98 (34.3)	490 (34.3)
⩾70	365 (14.6)	1825 (14.6)	49 (17.1)	245 (17.1)
Median	57.5	57.5	60.5	60.5
				
*Highest achieved education level*				
9 years comprehensive school or less	1457 (58.3)	5916 (47.4)	165 (57.7)	621 (43.4)
Upper secondary school (10–12 years)	756 (30.3)	4316 (34.6)	76 (26.6)	542 (37.9)
University education (>12 years)	254 (10.2)	2111 (16.9)	42 (14.7)	246 (17.2)
Missing	31 (1.2)	147 (1.2)	3 (1.1)	21 (1.5)
				
*Highest achieved occupational class*				
Manual workers	697 (27.9)	2952 (23.6)	90 (31.5)	350 (24.5)
Non-manual workers	1136 (45.5)	6224 (49.8)	125 (43.7)	697 (48.7)
Professionals	104 (4.2)	900 (7.2)	14 (4.9)	98 (6.9)
Farmers	99 (4.0)	570 (4.6)	17 (5.9)	60 (4.2)
Self-employed	142 (5.7)	845 (6.8)	18 (6.3)	105 (7.3)
Other	320 (12.8)	999 (8.0)	22 (7.7)	120 (8.4)
Total	2498 (100)	12 490 (100)	286 (100)	1430 (100)

**Table 2:** Association of parity with risk of non-cardia stomach cancer among Swedish women^a^

	**All non-cardia stomach cancer**		**Premenopausal cancer^b^**		**Postmenopausal cancer^b^**	
**Reproductive variables**	**Cases/controls**	**Odds ratio (95% CI)**	**Cases/controls**	**Odds ratio (95% CI)**	**Cases/controls**	**Odds ratio (95% CI)**
*All women*						
Nulliparous	335/1720	Reference	117/512	Reference	218/1208	Reference
Parous	2163/10 770	1.01 (0.89–1.15)	611/3128	0.82 (0.65–1.03)	1552/7642	1.11 (0.94–1.30)
						
*Ever-parous women*						
*Number of children*^c^						
1 child	481/2199	Reference	114/566	Reference	367/1633	Reference
>1 child	1682/8571	0.90 (0.80–1.02)	497/2562	0.96 (0.75–1.23)	1185/6009	0.88 (0.76–1.01)
2 children	905/4734	0.91 (0.80–1.03)	268/1444	0.95 (0.73–1.23)	637/3290	0.88 (0.76–1.02)
3 children	484/2549	0.87 (0.75–1.01)	160/786	0.97 (0.73–1.30)	324/1763	0.81 (0.68–0.96)
⩾4 children	293/1288	0.96 (0.81–1.15)	69/332	0.90 (0.62–1.29)	224/956	0.95 (0.77–1.16)
*P* for trend		0.40		0.74		0.34
						
*Age at first birth*^d^						
<21 years	648/2776	Reference	174/810	Reference	474/1966	Reference
21–24 years	731/3628	0.95 (0.84–1.07)	207/1052	1.09 (0.86–1.39)	524/2576	0.90 (0.78–1.04)
25–27 years	383/2125	0.91 (0.78–1.05)	123/593	1.29 (0.97–1.72)	260/1532	0.79 (0.67–0.95)
⩾28 years	401/2241	0.93 (0.80–1.09)	107/673	1.09 (0.81–1.48)	294/1568	0.88 (0.74–1.06)
*P* for trend		0.35		0.46		0.11
						
*Married women*						
*All married women*						
Nulliparous	53/430	Reference	19/153	Reference	34/295	Reference
Parous	772/4849	1.11 (0.78–1.59)	198/1428	0.75 (0.38–1.45)	574/3421	1.27 (0.83–1.95)
						
*Parous married women*						
1 Child	148/880	Reference	26/198	Reference	122/682	Reference
>1 Child	624/3969	0.94 (0.73–1.20)	172/1230	1.07 (0.59–1.94)	452/2739	0.90 (0.69–1.18)

CI=confidence interval.

a Adjusted for occupational class and education level.

b Attained age ⩾50 years was used to define postmenopausal women.

c Adjusted for occupational class, education level, and age at first birth.

d Adjusted for occupational class, education level, and the number of children.

**Table 3:** Association of parity with risk of cardia cancer among Swedish women^a^

	**All cardia stomach cancer**		**Premenopausal cancer^b^**		**Postmenopausal cancer^b^**	
**Reproductive variables**	**Cases/controls**	**Odds ratio (95% CI)**	**Cases/controls**	**Odds ratio (95% CI)**	**Cases/controls**	**Odds ratio (95% CI)**
*All women*						
Nulliparous	46/192	Reference	7/38	Reference	39/154	Reference
Parous	240/1238	0.7 (0.5–1.1)	53/262	0.9 (0.4–2.3)	187/976	0.7 (0.5–1.0)
						
*Ever-parous women*						
*Number of children*^c^						
1 child	53/227	Reference	15/49	Reference	38/178	Reference
>1 child	187/1011	0.7 (0.5–0.95)	38/213	0.5 (0.3–1.1)	149/798	0.7 (0.5–1.1)
2 children	87/570	0.6 (0.4–0.9)	25/118	0.7 (0.3–1.5)	62/452	0.6 (0.4–0.9)
3 children	62/292	0.8 (0.5–1.2)	9/59	0.3 (0.1–0.9)	53/233	0.9 (0.6–1.5)
⩾4 children	38/149	0.9 (0.5–1.5)	4/36	0.4 (0.1–1.6)	34/113	1.1 (0.6–2.0)
*P* for trend		0.97		0.04		0.31
						
*Age at first birth*^d^						
<21 years	72/317	Reference	11/67	Reference	61/250	Reference
21–24 years	77/411	0.9 (0.6–1.4)	23/81	1.9 (0.7–4.7)	54/330	0.8 (0.5–1.2)
25–27 years	54/248	1.2 (0.8–1.9)	11/53	1.5 (0.5–4.3)	43/195	1.2 (0.7–2.0)
⩾28 years	37/262	0.8 (0.5–1.4)	8/61	0.9 (0.3–3.0)	29/201	0.8 (0.5–1.5)
*P* for trend		0.71		0.72		0.94
						
*Married women*						
*All married women*						
Nulliparous	12/53	Reference	0/5	Reference	12/48	Reference
Parous	89/597	0.4 (0.2–0.9)	18/136	Not applicable	71/461	0.3 (0.1–0.9)
						
*Parous married women*						
1 child	14/87	Reference	2/13	Reference	12/74	Reference
>1 child	75/510	0.8 (0.3–1.8)	16/123	0.6 (0.1–4.2)	59/387	0.8 (0.3–2.0)

CI=confidence interval.

a Adjusted for occupational class and education level.

b Attained age ⩾50 years was used to define postmenopausal women.

c Adjusted for occupational class, education level, and age at first birth.

d Adjusted for occupational class, education level, and the number of children.

**Table 4:** Summary of previous studies on the association between reproductive factors and stomach cancer risk

**Authors (year)**	**Study design**	**Study population**	**Study period (country)**	**Exposure (reference group)**	**Estimate of relative risk for stomach cancer**
Frise *et al* (2006)	Population-based case–control study	326 cases and 326 age-matched controls	1995–1997 (Canada)	>3 pregnancies (nulliparity)	Overall, 0.56 (0.32–0.99) Cardia, 0.51 (0.18–1.43) Non-cardia, 0.71 (0.37–1.36)
(Kaneko *et al* (2003)	Prospective cohort study	40 535 postmenopausal women, 156 deaths due to stomach cancer	1988–1997 (Japan)	Ever pregnant (never)	Overall, 0.62 (0.27–1.41)
Inoue *et al* (2002)	Hospital-based case–control study	365 postmenopausal cases and 1825 age-matched controls	1988–1998 (Japan)	Ever pregnant (never)	Overall, 1.13 (0.75–1.70)
Palli *et al* (1994)	Population-based case–control study	339 postmenopausal cases and 515 age-matched controls	1985–1987 (Italy)	Ever pregnant (never)	Overall, 1.0 (0.6–1.5)
La Vecchia *et al* (1994)	Hospital-based case–control study	229 postmenopausal cases and 614 controls	1985–1993 (Italy)	>3 pregnancies (nulliparity)	Overall, 1.9 (1.0–3.5)
Kvale *et al* (1994)	Prospective cohort study	61 774 women, 492 cases	1961–1980 (Norway)	>4 pregnancies among women <50 years old at the start of follow-up (nulliparity)	Overall, 1.12 (*P* for trend=0.71)
Plesko *et al* (1985)	Population-based case–control study	3613 deaths due to stomach cancer, 182 415 deaths due to all causes	1968–1977 (Slovakia)	>5 pregnancies (nulliparity)	Overall, 1.34 (*P* for trend <0.001)
				>5 pregnancies (one pregnancy)	Overall, 1.21 (*P* for trend <0.01)
Miller *et al* (1980)	Population-based case–control study	260 stomach cancer cases	1969–1971 (Canada)	>3 pregnancies (nulliparity)	Overall, 1.59 (*P* for trend=0.05)

## References

[bib1] Cunningham FG, Gant NF, Leveno KL, Gilstrap III LC, Hauth JC, Wenstrom KD (2001) Williams Obstetrics. McGraw-Hill: New York

[bib2] Ekstrom AM, Signorello LB, Hansson LE, Bergstrom R, Lindgren A, Nyren O (1999) Evaluating gastric cancer misclassification: a potential explanation for the rise in cardia cancer incidence. J Natl Cancer Inst 91: 786–790 10328109 10.1093/jnci/91.9.786

[bib3] Frise S, Kreiger N, Gallinger S, Tomlinson G, Cotterchio M (2006) Menstrual and reproductive risk factors and risk for gastric adenocarcinoma in women: findings from the Canadian National Enhanced Cancer Surveillance System. Ann Epidemiol 16: 908–916 16843679 10.1016/j.annepidem.2006.03.001

[bib4] Furukawa H, Iwanaga T, Koyama H, Taniguchi H (1982a) Effect of sex hormones on carcinogenesis in the stomachs of rats. Cancer Res 42: 5181–5182 7139622

[bib5] Furukawa H, Iwanaga T, Koyama H, Taniguchi H (1982b) Effect of sex hormones on the experimental induction of cancer in rat stomach – a preliminary study. Digestion 23: 151–155 7106416 10.1159/000198722

[bib6] Gold EB, Bromberger J, Crawford S, Samuels S, Greendale GA, Harlow SD, Skurnick J (2001) Factors associated with age at natural menopause in a multiethnic sample of midlife women. Am J Epidemiol 153: 865–874 11323317 10.1093/aje/153.9.865

[bib7] Gram IT, Braaten T, Terry PD, Sasco AJ, Adami HO, Lund E, Weiderpass E (2005) Breast cancer risk among women who start smoking as teenagers. Cancer Epidemiol Biomarkers Prev 14: 61–66 15668477

[bib8] Inoue M, Ito LS, Tajima K, Yamamura Y, Kodera Y, Takezaki T, Hamajima N, Hirose K, Kuroishi T, Tominaga S (2002) Height, weight, menstrual and reproductive factors and risk of gastric cancer among Japanese postmenopausal women: analysis by subsite and histologic subtype. Int J Cancer 97: 833–838 11857364 10.1002/ijc.10149

[bib9] Kaneko S, Tamakoshi A, Ohno Y, Mizoue T, Yoshimura T (2003) Menstrual and reproductive factors and the mortality risk of gastric cancer in Japanese menopausal females. Cancer Causes Control 14: 53–59 12708725 10.1023/a:1022596104796

[bib10] Kvale G, Heuch I, Nilssen S (1994) Parity in relation to mortality and cancer incidence: a prospective study of Norwegian women. Int J Epidemiol 23: 691–699 8002181 10.1093/ije/23.4.691

[bib11] La Vecchia C, D'Avanzo B, Franceschi S, Negri E, Parazzini F, Decarli A (1994) Menstrual and reproductive factors and gastric-cancer risk in women. Int J Cancer 59: 761–764 7989115 10.1002/ijc.2910590609

[bib12] Linne Y, Barkeling B, Rossner S (2002) Long-term weight development after pregnancy. Obes Rev 3: 75–83 12120423 10.1046/j.1467-789x.2002.00061.x

[bib13] Luoto R, Kaprio J, Uutela A (1994) Age at natural menopause and sociodemographic status in Finland. Am J Epidemiol 139: 64–76 8296776 10.1093/oxfordjournals.aje.a116936

[bib14] Matsuyama S, Ohkura Y, Eguchi H, Kobayashi Y, Akagi K, Uchida K, Nakachi K, Gustafsson JA, Hayashi S (2002) Estrogen receptor beta is expressed in human stomach adenocarcinoma. J Cancer Res Clin Oncol 128: 319–324 12073050 10.1007/s00432-002-0336-3 PMC12164475

[bib15] Mattsson B, Wallgren A (1984) Completeness of the Swedish Cancer Register. Non-notified cancer cases recorded on death certificates in 1978. Acta Radiol Oncol 23: 305–313 6095600 10.3109/02841868409136026

[bib16] Miller AB, Barclay TH, Choi NW, Grace MG, Wall C, Plante M, Howe GR, Cinader B, Davis FG (1980) A study of cancer, parity and age at first pregnancy. J Chronic Dis 33: 595–605 7410520 10.1016/0021-9681(80)90002-8

[bib17] Nyren O, Adami H-O (2002) Stomach cancer. In Textbook of Cancer Epidemiology, Adami H-O, Hunter D, Trichopoulos D (eds), pp 171–176. Oxford University Press: New York

[bib18] Palli D, Cipriani F, Decarli A, Galli M, Saieva C, Fraumeni Jr JF, Blot WJ, Buiatti E (1994) Reproductive history and gastric cancer among post-menopausal women. Int J Cancer 56: 812–815 8119771 10.1002/ijc.2910560609

[bib19] Parkin DM, Bray F, Ferlay J, Pisani P (2005) Global cancer statistics, 2002. CA Cancer J Clin 55: 74–108 15761078 10.3322/canjclin.55.2.74

[bib20] Plesko I, Preston-Martin S, Day NE, Tzonou A, Dimitrova E, Somogyi J (1985) Parity and cancer risk in Slovakia. Int J Cancer 36: 529–533 4055127 10.1002/ijc.2910360502

[bib21] Rodstrom K, Bengtsson C, Milsom I, Lissner L, Sundh V, Bjourkelund C (2003) Evidence for a secular trend in menopausal age: a population study of women in Gothenburg. Menopause 10: 538–543 14627863 10.1097/01.GME.0000094395.59028.0F

[bib22] Singh S, Poulsom R, Wright NA, Sheppard MC, Langman MJ (1997) Differential expression of oestrogen receptor and oestrogen inducible genes in gastric mucosa and cancer. Gut 40: 516–520 9176081 10.1136/gut.40.4.516 PMC1027128

[bib23] Sipponen P, Correa P (2002) Delayed rise in incidence of gastric cancer in females results in unique sex ratio (M/F) pattern: etiologic hypothesis. Gastric Cancer 5: 213–219 12491079 10.1007/s101200200037

[bib24] Taylor AH, Al-Azzawi F (2000) Immunolocalisation of oestrogen receptor beta in human tissues. J Mol Endocrinol 24: 145–155 10657006 10.1677/jme.0.0240145

[bib25] Wu CW, Chi CW, Chang TJ, Lui WY, P'Eng FK (1990) Sex hormone receptors in gastric cancer. Cancer 65: 1396–1400 2306686 10.1002/1097-0142(19900315)65:6<1396::aid-cncr2820650625>3.0.co;2-2

